# The anterolateral triangle as window on the foramen lacerum from transorbital corridor: anatomical study and technical nuances

**DOI:** 10.1007/s00701-023-05704-5

**Published:** 2023-07-21

**Authors:** Sergio Corvino, Daniele Armocida, Martina Offi, Giovanni Pennisi, Benedetta Burattini, Andres Villareal Mondragon, Felice Esposito, Luigi Maria Cavallo, Matteo de Notaris

**Affiliations:** 1 Division of Neurosurgery, Department of Neuroscience and Reproductive and Odontostomatological Sciences, Università Degli Studi Di Napoli Federico II, 80131 Naples, Italy; 2 PhD Program in Neuroscience, Department of Neuroscience and Reproductive and Odontostomatological Sciences, Università Degli Studi Di Napoli Federico II, 80131 Naples, Italy; 3 Neurosurgery Division, Human Neurosciences Department, “Sapienza” University, 00185 Rome, Italy; 4 Institute of Neurosurgery, Fondazione Policlinico Universitario A. Gemelli, Rome, Italy - Division of Neurosurgery, Catholic University of Rome, Rome, Italy; 5 Clinica Imbanaco Grupo Quiron Salud, Universidad del Valle, Cali, Colombia; 6 Department of Neuroscience, Neurosurgery Operative Unit, “San Pio” Hospital, 82100 Benevento, Italy; 7 Laboratory of Neuroanatomy, EBRIS Foundation, European Biomedical Research Institute of Salerno, Salerno, Italy

**Keywords:** Endoscopic transorbital, Foramen lacerum, Middle fossa triangles, Meckel’s cave, Vidian nerve

## Abstract

**Objective:**

Neurosurgical indications for the superior eyelid transorbital endoscopic approach (SETOA) are rapidly expanding over the last years. Nevertheless, as any new technique, a detailed knowledge of the anatomy of the surgical target area, the operative corridor, and the specific surgical landmark from this different perspective is required for a safest and successful surgery. Therefore, the aim of this study is to provide, through anatomical dissections, a detailed investigation of the surgical anatomy revealed by SETOA via anterolateral triangle of the middle cranial fossa. We also sought to define the relevant surgical landmarks of this operative corridor.

**Methods:**

Eight embalmed and injected adult cadaveric specimens (16 sides) underwent dissection and exposure of the cavernous sinus and middle cranial fossa via superior eyelid endoscopic transorbital approach. The anterolateral triangle was opened and its content exposed. An extended endoscopic endonasal trans-clival approach (EEEA) with exposure of the cavernous sinus content and skeletonization of the paraclival and parasellar segments of the internal carotid artery (ICA) was also performed, and the anterolateral triangle was exposed. Measurements of the surface area of this triangle from both surgical corridors were calculated in three head specimens using coordinates of its borders under image-guide navigation.

**Results:**

The drilling of the anterolateral triangle via SETOA unfolds a space that can be divided by the course of the vidian nerve into two windows, a wider “supravidian” and a narrower “infravidian,” which reveal different anatomical corridors: a “medial supravidian” and a “lateral supravidian,” divided by the lacerum segment of the ICA, leading to the lower clivus, and to the medial aspect of the Meckel’s cave and terminal part of the horizontal petrous ICA, respectively. The infravidian corridor leads medially into the sphenoid sinus. The arithmetic means of the accessible surface area of the anterolateral triangle were 45.48 ± 3.31 and 42.32 ± 2.17 mm^2^ through transorbital approach and endonasal approach, respectively.

**Conclusion:**

SETOA can be considered a minimally invasive route complementary to the extended endoscopic endonasal approach to the anteromedial aspect of the Meckel’s cave and the foramen lacerum. The lateral loop of the trigeminal nerve represents a reliable surgical landmark to localize the lacerum segment of the ICA from this corridor. Nevertheless, as any new technique, a learning curve is needed, and the clinical feasibility should be proven.

## Introduction

The superior eyelid endoscopic transorbital approach (SETOA), initially adopted mainly by ophthalmologists for orbital pathologies, is rapidly increasing in popularity among neurosurgeons over the last years, as witnessed by the numerous and heterogeneous anatomical studies and published surgical series concerning intracranial neurosurgical pathologies extending from the anterior skull base to the petrous apex[7, 11, 12, 26, 30, 31, 46]. Indeed, this corridor allows the direct access to the ventral paramedian and lateral aspects of the anterior and middle cranial fossae, in a minimally invasive fashion, with reduced bone destruction, minimal or no brain retraction, no manipulation of neurovascular structures, satisfactory esthetic results, and short hospital stay[3, 8, 31].

Nevertheless, as any new technique, its surgical potential, in terms of exposure and access areas, well-defined anatomical landmarks, pros and cons, pathologies, and patients’ features suitable for this technique, as well as its therapeutic and/or diagnostic role, are being explored. In addition, a learning curve[12], which first of all includes a detailed knowledge of the anatomy of the surgical target area, the operative corridor and the specific surgical landmarks from this different perspective, is required for a safest and successful surgery.

Several technical variants and anatomical corridors revealed by this approach are continuously described[29, 32, 46] with the aim of reaching even more different surgical target areas and planning optimal and tailored-surgical approaches accordingly.

In this scenario, we focused on the anterolateral triangle of the middle fossa as a front-door to the region of foramen lacerum, because of the anatomical and functional inherent advantages which it offers and make it a relatively safe surgical corridor: it comes into endoscopic view immediately after interperiosteal-dural dissection, and it is devoid of vital or highly functional neurovascular structures.

Therefore, this study provides, through anatomical dissections, a detailed investigation of the surgical anatomy revealed by the transorbital endoscopic approach through the anterolateral triangle of the middle cranial fossa, including the foramen lacerum and its adjacent structures. In addition, we sought to define the relevant key surgical landmarks of this operative corridor, also providing comparative surgical nuances with the endoscopic endonasal route to the same target area[5, 21, 22].

We consider that this operative corridor, in isolated or combined manner, may be suitable for several purposes, such as for diagnostic biopsies, gross total, or subtotal resection of lesions involving the medial aspect of the Meckel’s cave with lateral extension into the middle and infratemporal fossae.

## Materials and methods

Anatomical dissections were performed at the Laboratory of Skull Base and Micro-neurosurgery in the Weill Cornell Neurosurgical Innovations and Training Center, New York, USA, and EBRIS Laboratory of Neuroanatomy, Salerno, Italy. Eight adult cadaveric specimens (10 sides), embalmed and injected with red and blue latex for the arteriosus and venous blood vessels, respectively, were adequately secured in a rigid three-pin fixation and underwent endoscopic transorbital approach bilaterally, first, and extended endoscopic endonasal transclival approach, then, for a total of 24 surgical procedures (8 EEEA, 16 SETOA). After the initial step under macroscopic visualization, SETOA was performed with a 4 mm in diameter and 18 cm in length, rigid endoscope as optical device, with 0° and 30° rod lenses (Karl Storz, Tuttlingen, Germany), connected to a light source (300 W Xenon, Karl Storz) through a fiberoptic cable and to an HD camera (Endovision Telecam SL; Karl Storz). The entire endonasal transclival approach was a purely endoscopic procedure.

A high-resolution CT scan was performed in 3 head specimens before the dissections and data were uploaded into a neuronavigation system (Brainlab cranial navigation system). Quantitative analysis of the accessible surface area of the anterolateral triangle exposed through each approach was calculated.

### Superior eyelid transorbital endoscopic approach (SETOA) to the middle cranial fossa

A SETOA to the middle cranial fossa was performed as previously reported in the pertinent literature[11].

The head specimen was placed in supine and neutral position, 10° flexed and 5° contralaterally rotated.

**Fig. 1:**
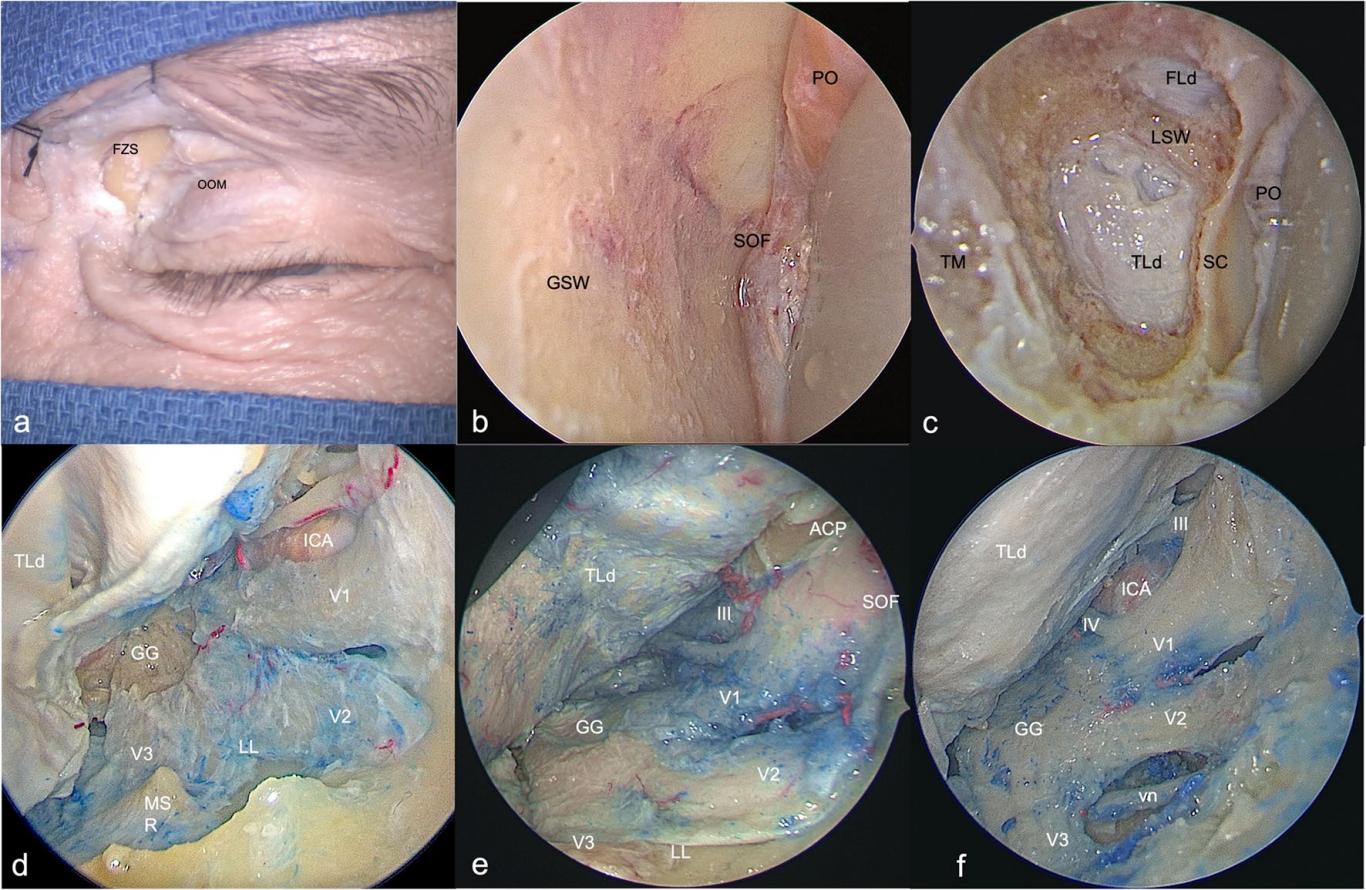
Superior eyelid transorbital endoscopic approach (SETOA) to the middle cranial fossa (right side).** a** Skin incision on a wrinkle of the upper eyelid and carried in the depth through the orbicularis oculi muscle (OOM) and extended laterally until to expose the fronto-zygomatic suture (FZS); **b** after the subperiosteal/periorbital dissection was performed up to identify the superior (SOF) and inferior orbital fissures, the corridor between the periorbit (PO) content medially and the greater sphenoid wing (GSW) forming the lateral wall of the orbit, laterally, is exposed; **c** exposure of the temporal lobe pole dura mater (TLd) and the sagittal crest (SC) after the drilling of the lateral wall of the orbit; **d** interperiosteal/dural dissection of the cavernous sinus lateral wall and of the middle fossa with identification of the midsubtemporal ridge (MSR) at the base of the anterolateral triangle between the foramina rotundum and ovale; **e** flattening of the middle fossa floor and exposure of the III and IV cranial nerves, the trigeminal nerve with its ganglion (GG), branches (V1, V2, V3), and lateral loop (LL) inside the periosteal layer; **f** drilling of the anterolateral triangle with skeletonization of the vidian nerve (vn). (FZS, fronto-zygomatic suture; OOM, orbicularis oculi muscle; GSW, greater sphenoid wing; SOF, superior orbital fissure; PO, periorbit; TLd, temporal lobe dura; SC, sagittal crest; GG, gasserian ganglion; ACP, anterior clinoid process; LL, lateral loop, MSR, mid-subtemporal ridge)

A skin incision was placed in a superior eyelid wrinkle, and once the orbicularis oculi muscle was cut taking care not to violate the fibers of the levator palpebrae, the dissection was carried in depth up to the superior orbital rim and extended laterally up to the fronto-zygomatic suture. After cutting the periosteum where it became continuous with the periorbita, the dissection continued, with endoscopic assistance, in a subperiosteum/periorbital plane within the orbit until the lateral margin of the inferior and superior orbital fissures. Zygomatico-facial and zygomatico-temporal arteries were identified and cut. At that point, once the zygomatic body and the intraorbital part of the greater sphenoid wing including the sagittal crest[6] were drilled until to expose the temporal pole dura mater, an interperiosteal-dural dissection via meningo-orbital band (MOB)[10] was performed to unlock the lateral wall of the CS up to the gasserian ganglion (GG). After identification and cutting of the middle meningeal artery (MMA), the temporal pole was elevated in extradural fashion, and the peeling of the floor of the middle cranial fossa was completed. Once the midsubtemporal ridge[48] was identified and flattened laterally to the trigeminal nerve lateral loop — the angle described by the lateral margin of V2 and the ventral margin of V3 at the gasserian ganglion[48] — the drilling of the area of the skull base bounded by the lower border of V2, superiorly, the upper border of V3, inferiorly, and by the line which connects foramina rotundum and ovale, defining the anterolateral triangle of the middle cranial fossa[39], and the skeletonization of the vidian canal, completed the dissection procedure (Fig. 1). The exposure of the vidian nerve started by drilling immediately inferiorly to the foramen rotundum in lateral-to-medial and anterior-to-posterior directions to firstly identify the sphenoid sinus and the superior border of the vidian canal and then proceeded posteriorly along the lower and lateral borders of the same canal up to the trigeminal lateral loop and the lacerum foramen. The drilling should occur along the inferior hemicircumference of the vidian canal as the ICA is located along the superior border. Lastly, the skeletonization proceeded between the inferior border of V2 and the superior margin of the vidian canal.

### Extended endoscopic endonasal approach (EEEA) to the clivus

An extended endoscopic endonasal transsphenoidal approach was performed as previously reported in the literature[4, 5]. Conversely from the standard endoscopic endonasal transsphenoidal approach, to obtain a wider exposition of the CS, the sphenoidotomy was extended more laterally, and the posterior ethmoidal cells were opened. Furthermore, to expand the operative corridor, the uncinate process was removed, and the bulla ethmoidalis was opened, thus allowing to reach and remove the anterior ethmoid cells. The removal of the posterior ethmoid cells and the anterior wall of the sphenoid sinus allowed to expose the lateral wall of the sphenoid sinus with a direct trajectory, and once it was removed, the CS came into the view. Lastly, the course of the vidian nerve allowed us to reach the lacerum ICA.

### Quantitative analysis

The area of the anterolateral triangle exposed through each approach was calculated using the Brainlab cranial navigation system. For each approach, 3 points — foramen rotundum, foramen ovale and trigeminal nerve lateral loop — corresponding to 3D coordinates obtained by using the stereotactic image-guidance system (Brainlab cranial navigation system), were used as landmark limits of the triangle.

**Fig. 2:**
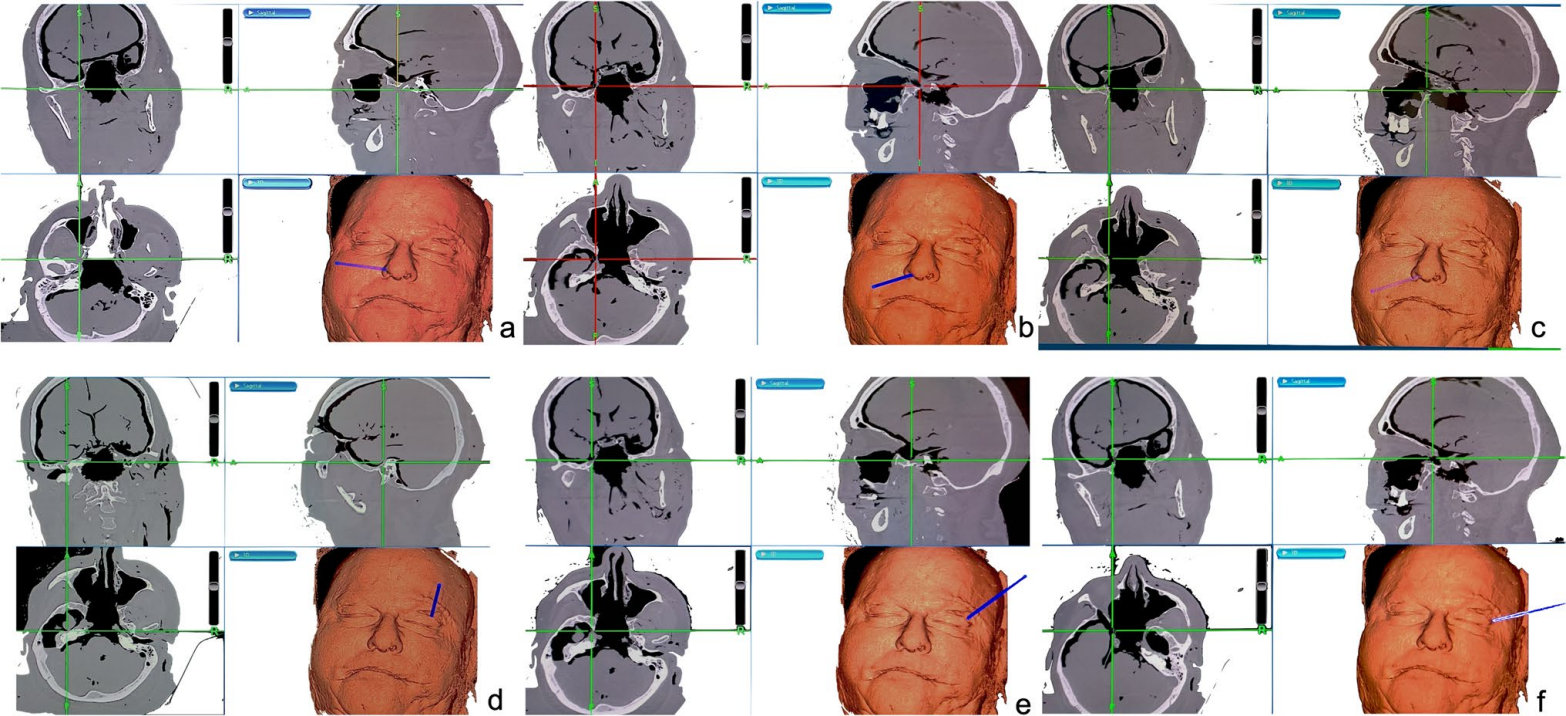
Imagine guidance from neuronavigation system showing measurements of the anterolateral triangle vertices from endonasal (**a**–**c**) and transorbital perspectives (**d**–**f**). Foramen Rotundum from endonasal (**a**) and transorbital (**d**) perspectives; foramen ovale from endonasal (**b**) and transorbital (**e**) perspectives; lateral loop of trigeminal nerve from endonasal (**c**) and transorbital (**f**) perspectives

Points registered were expressed as Cartesian coordinates (*x*, *y*, *z*) on the Brainlab workstation. Each point was acquired 3 times, the arithmetic mean for each coordinate was calculated, and a scalene triangle was generated, whose surface area was calculated using the predetermined references at the borders of the triangle which were marked using the navigation device (Fig. 2).

### Statistical analysis

A *Student’s t test* was used to compare the mean surface area of the anterolateral triangle of the middle fossa exposed through transorbital and endonasal corridors. A *p* value ≤ 0.05 was considered statistically significant.

## Results

**Fig. 3:**
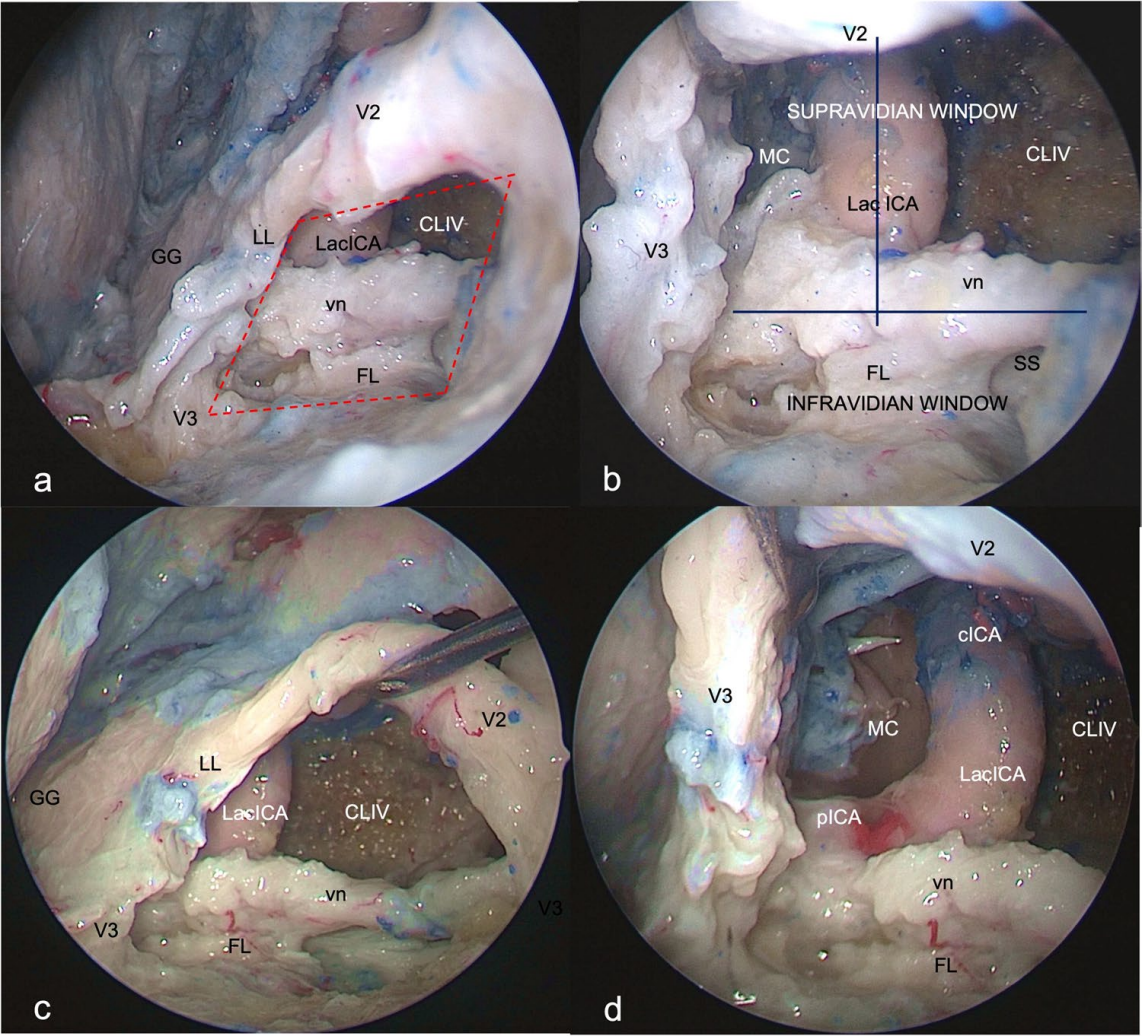
Exposure of the content of the anterolateral triangle of the middle cranial fossa through SETOA (right side). **a** Identification of a quadrangular space (red dotted line) and its content (lacerum ICA, vidian nerve, foramen lacerum, lower clivus, medial aspect of Meckel’s cave) limited by the inferior border of V2, superiorly, the superior border of V3, posteriorly, the line crossing the most anterior limit of exposure of the vidian nerve and joining the foramen rotundum and the point where the greater wing joints the body of the sphenoid bone, anteriorly, and the line connecting this last point and the foramen ovale inferiorly; **b** identification of the supravidian and infravidian windows, divided by the course of the vidian nerve from its distal end of exposure up to its disappearing behind V3, and of the related disclosed corridors: the “medial supravidian” and the “lateral supravidian,” divided by the lacerum segment of ICA, and leading to the lower clivus, and to the medial aspect of the Meckel’s cave and to the distal end of the horizontal segment of the petrous ICA, respectively; **c** expanded view of the *“*medial supravidian corridor” after gentle upward displacement of V2;** d** expanded view of the “lateral supravidian corridor” after gentle lateralization of the gasserian ganglion. (GG, gasserian ganglion; Lac ICA, lacerum internal carotid artery; CLIV, clivus; FL, foramen lacerum; vn, vidian nerve; MC, Meckel’s cave; cICA, cavernous internal carotid artery; pICA, petrous internal carotid artery; LL, lateral loop)

The opening of the anterolateral triangle of the middle cranial fossa through transorbital endoscopic approach allowed us to discover the vidian nerve (vn) and artery in the homonymous canal along their course until the upper part of the anterolateral edge of the foramen lacerum (FL), where the posterior opening of the canal is filled with cartilaginous tissue that blends into the more medially positioned cartilage that fills the foramen lacerum, across the “mandibular strut”[47]. The lacerum segment of the ICA, at its transition zone from the horizontal petrous segment to the posterior ascending cavernous segment, medially to the petrolingual ligament (PL), and the associated carotid sympathetic plexus, could be also exposed (Fig. 3). It was possible to appreciate a space (red dotted lines, Fig. 3a) — quadrangular in shape in 6 out of eight specimens — bounded by the lower border of V2, superiorly, the upper border of V3, posteriorly, by the line crossing the most anterior limit of exposure of the vidian nerve and joining the foramen rotundum and the point where the greater wing joints the body of the sphenoid bone, anteriorly, and the line between the latter point and the foramen ovale posteriorly. The anteroinferior point of this space can be always identified, but it is not a fixed point, and its position depends on the degree of medial retraction of the orbital content; as result, the unfolded space is not always quadrangular in shape.

This space allowed us to distinguish two windows divided by the course of the vidian nerve until the point where it blends into the cartilaginous tissue of the FL and which revealed different corridors (Fig. 3):

- A wider superior window (“supravidian”), which unfolded two corridors divided by the lacerum segment of the ICA: a “medial supravidian corridor,” which leaded in a more lateral-to-medial direction to the lower clivus and which could be expanded after a gentle upward displacement of V2, and a “lateral supravidian corridor” which leaded to the medial aspect of the Meckel’s cave and the terminal portion of the horizontal segment of the petrous ICA (pICA) after gentle lateralization of the gasserian ganglion (GG).
- A narrower inferior window (“infravidian”), which revealed the lower intracranial part of the foramen lacerum, distally, and the sphenoid sinus proximally and medially.

In all, 8 specimens undergone dissection, and thus in sixteen, transorbital procedures were performed; the lacerum segment of the ICA medially to the distal end of vidian nerve and petrolingual ligament was identified in the depth of the corner between the origin of V2 and V3 from the gasserian ganglion, also known as trigeminal “lateral loop,” and thus at the postero-superior corner of the identified space, along the latero-to-medial trajectory of the transorbital approach.

### Quantitative analysis

The arithmetic means of the accessible area of the anterolateral triangle were 45.48 ± 3.31 mm^2^ and 42.32 ± 2.17 mm^2^ through transorbital approach and endonasal approach, respectively.

## Discussion

The anterolateral triangle of the middle fossa is bounded by the lower border of V2 superiorly, the upper border of V3 inferiorly, and by the line joining the foramina rotundum and ovale anteriorly[39]. Since its first description by Dolenc in 1989[14], while there is unanimous consent on its anatomical limits, there is not agreement on its nomenclature. Furthermore, albeit the size of its drilling is variable according to the natural anatomic variability among patients in physiological conditions, some quantitative anatomic studies, including the present one, clearly described its mean surface area from different surgical perspectives[13, 17, 19, 49] (Table 1): that area in the present study resulted 45.48 ± 3.31 mm^2^ from transorbital route versus 42.32 ± 2.17 mm^2^ from endonasal route, with no statistically significant difference between them (*p* = 0.08).

**Table 1:** Comparison of mean accessible surface area of the anterolateral triangle through different operative corridors with data from the literature

Surgical route	Authors and Year	Anterolateral triangle accessible area (mean ± SD mm^2^)
Microsurgical transcranial	- Watanabe et al.[49] 2003	49.8 ± 15
	- Isolan et al.[19] 2007	51.52 ± 4.25
	- Granger et al.[17] 2018	20.46 ± 9.3
Endoscopic endonasal	- Dolci et al.[13] 2016	47.27 ± 5.27
	- Present study	42.32 ± 2.17
Endoscopic transorbital	- Present study	45.48 ± 3.31

To the best of our knowledge, the present study is the first one to measure through transorbital corridor the accessible surface area of the anterolateral triangle. These values can variously modify in pathologic conditions, following the primarily or secondary involvement of the parasellar region and displacement of second and third branches of the trigeminal nerve.

When opened from transcranial microsurgical route, mainly through the fronto-temporo-orbito-zygomatic or sub-temporal approaches, the lateral-to-medial trajectory allows the exposure of the vidian canal and its content, namely the vidian nerve and artery and the sphenoid sinus[39].

The opening of the anterolateral triangle through EEEA provides the visualization of the inferomedial temporal dural of the middle cranial fossa[13, 24]. This corridor allows the resection of lesions of the cavernous sinus with anterolateral extension or pituitary adenomas thanks to their softer consistency and so easier to suction out; in addition, this triangle is used to gain access to critical structures, such as anterolateral aspect of the C4 segment of the ICA and the origin of the inferolateral trunk[17].

The drilling of the anterolateral triangle through SETOA ensured the exposure of the same anatomical structures of the transcranial route, but thanks to its anterior-to-posterior and lateral-to-medial trajectories and the capability of the endoscope to bring the eyes of the surgeon close to the surgical field, this approach allowed us to follow the course of the vidian nerve more in the depth, posteriorly, and in a specular direction to it until the lacerum foramen and the related ICA segment and also to explore this area and the adjacent structures. In detail, we identified a “supravidian” window which revealed two different corridors divided by the lacerum segment of the ICA: a “medial supravidian” leading to the lower clivus and which could be expanded by the gentle upward displacement of V2 and a “lateral supravidian” leading to the medial aspect of the Meckel’s cave and the terminal portion of the horizontal petrous ICA and which could be expanded by the gentle lateralization of the gasserian ganglion. The “infravidian” window unfolded the sphenoid sinus and the lower intracranial part of the lacerum foramen, proximally and distally, respectively, along the surgical route.

Several lesions of the skull-base involving the region of the foramen lacerum, both as primary tumors such as chondrosarcomas, or secondary to pathologies affecting the cavernous sinus, Meckel’s cave, petrous apex, clival and petroclival regions, pterygopalatine, and infratemporal fossae, may require the exposure of the foramen lacerum and Meckel’s cave. As this area is hidden underneath the gasserian ganglion, its exposure through the traditional microsurgical approaches, usually classified in anterolateral, posterolateral, and lateral[18, 40, 42, 50], is difficult and requires the full mobilization and/or transection of V3 and gasserian ganglion[50]; indeed, all of these routes share the limit of being unable to access the medial aspect of Meckel’s cave. The access to the anteromedial aspect of the Meckel’s cave requires crossing the anteromedial and anterolateral triangles of the middle fossa[18, 27], but for lesions located posteriorly inside this dural pocket, the transgression of the trigeminal nerve is required.

The medial aspect of the Meckel’s cave, referred to as “quadrangular space” or “front door of Meckel’s cave” by Kassam et al.[22], is better exposed and without the need to the cross cranial nerves and vessels from the anteromedial corridor provided by the endoscopic endonasal approach[9, 16, 22, 34, 38, 43], but often at the expense of the integrity of the vidian nerve [1, 9, 22, 23, 51] and the related ophthalmic complications[36].

Among the several approaches, with related pro and cons, described for accessing Meckel’s cave[43], only two cadaveric laboratory studies have focused on the anatomical and technical implications of the lateral endoscopic orbital route to this area[15, 37], but having as target the antero-lateral and superior aspects of this dural recess. Jeon et al.[20]. report gross total resection in 7 out of 9 patients with Meckel’s cave disease who underwent isolated or combined endoscopic transorbital approach through anteromedial triangle or transorbital extended variant through the anterolateral triangle, with low rate of morbidities. Kong et al. [25] report the use of the anteromedial triangle of the cavernous sinus for type A, C, and D1 tumors according to Samii’s modified classification of trigeminal schwannoma [41] and the use of the anterolateral triangle for type D3 tumors with extension to the infratemporal fossa.

**Fig. 4:**
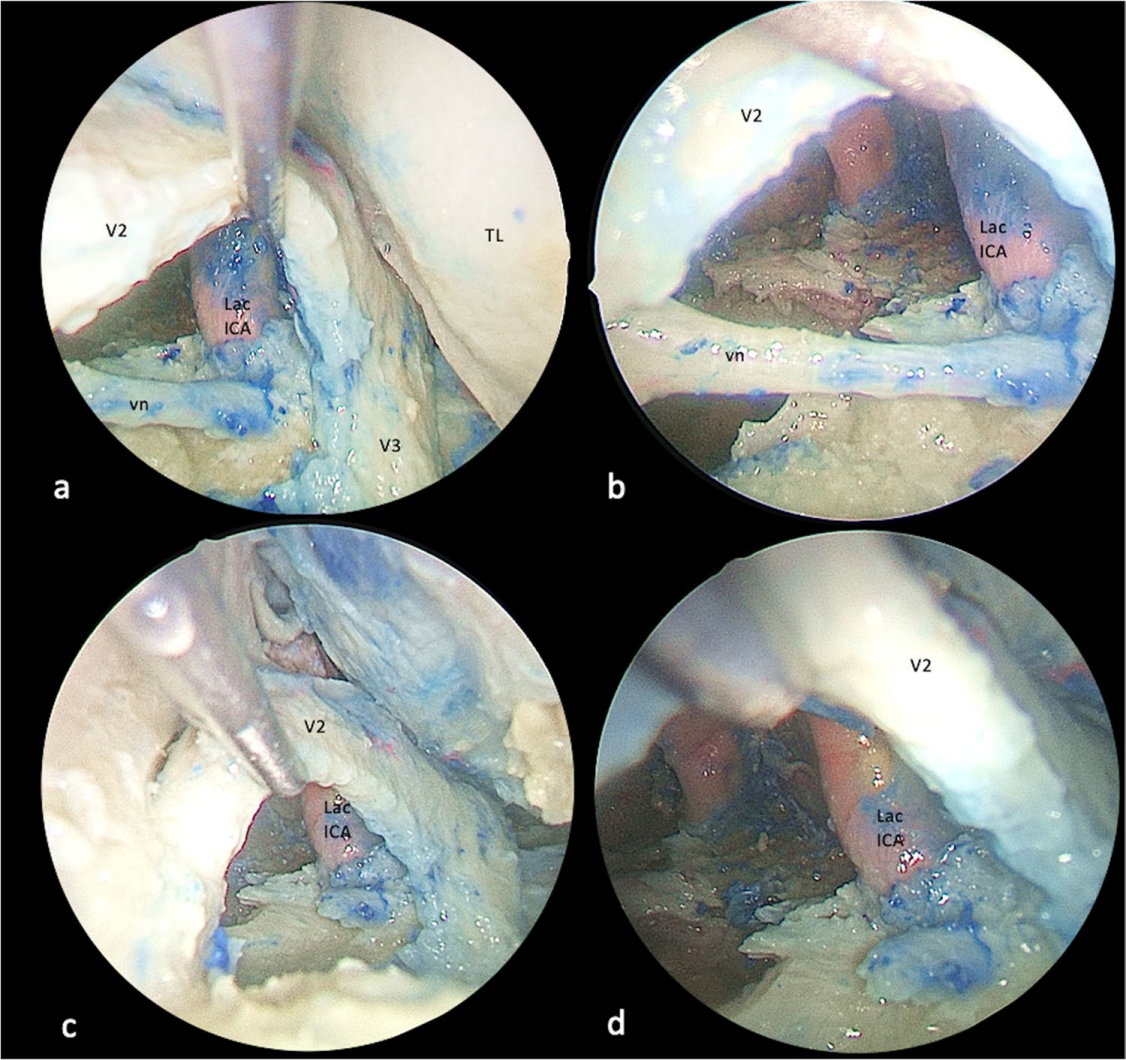
Exposure of the foramen lacerum region through endoscopic corridors before (**a**–**b**) and after (**c**–**d)** removal of the vidian nerve (left side). The exposure and working areas of the foramen lacerum, medial aspect of Meckel’s cave, and lower clivus increases after resection of the vidian nerve (**c**–**d**)

The present anatomical study investigates the transorbital endoscopic corridor to the foramen lacerum and the anteromedial aspect of Meckel’s cave via anterolateral triangle of the middle fossa without violating the integrity of cranial nerves. In our opinion, this pathway could be useful for several purposes: the diagnosis, through biopsy, of lesions of uncertain nature and whose radiologic features can mimic schwannoma, such as sarcoidosis, lymphoma, and inflammatory diseases and then for addressing their management through medical or oncological treatments while avoiding an alternative unnecessary and aggressive surgical approach[2]. Furthermore, this corridor can be adopted for the treatment of tumors like schwannomas of the trigeminal nerve and meningioma involving the medial aspect of the Meckel’s cave and with anterolateral extension, adjecting into the middle fossa, in which the vidian nerve is displaced but not enveloped by the lesion, in which the pattern of growth of the lesion expands the front-door to the transorbital corridor[35], or with downward extension into the infratemporal fossa, and/or when the goal of surgery is a subtotal extent of resection followed by adjuvant treatments. Again, the transorbital route will be very useful in a very small subset of patients which are affected by neurotrophic keratitis due to V1 injury, in which sparing the integrity of the vidian nerve is very important[1]. In contrast, if the vidian nerve is enveloped and not dissociable from the lesion, the sacrifice of the nerve will provide a wider surgical corridor to the foramen lacerum, the lower clivus, and the medial aspect of Meckel’s cave through fusion of the supra and infravidian windows, albeit at the expense of the ophthalmological complications (Fig. 4).

**Fig. 5:**
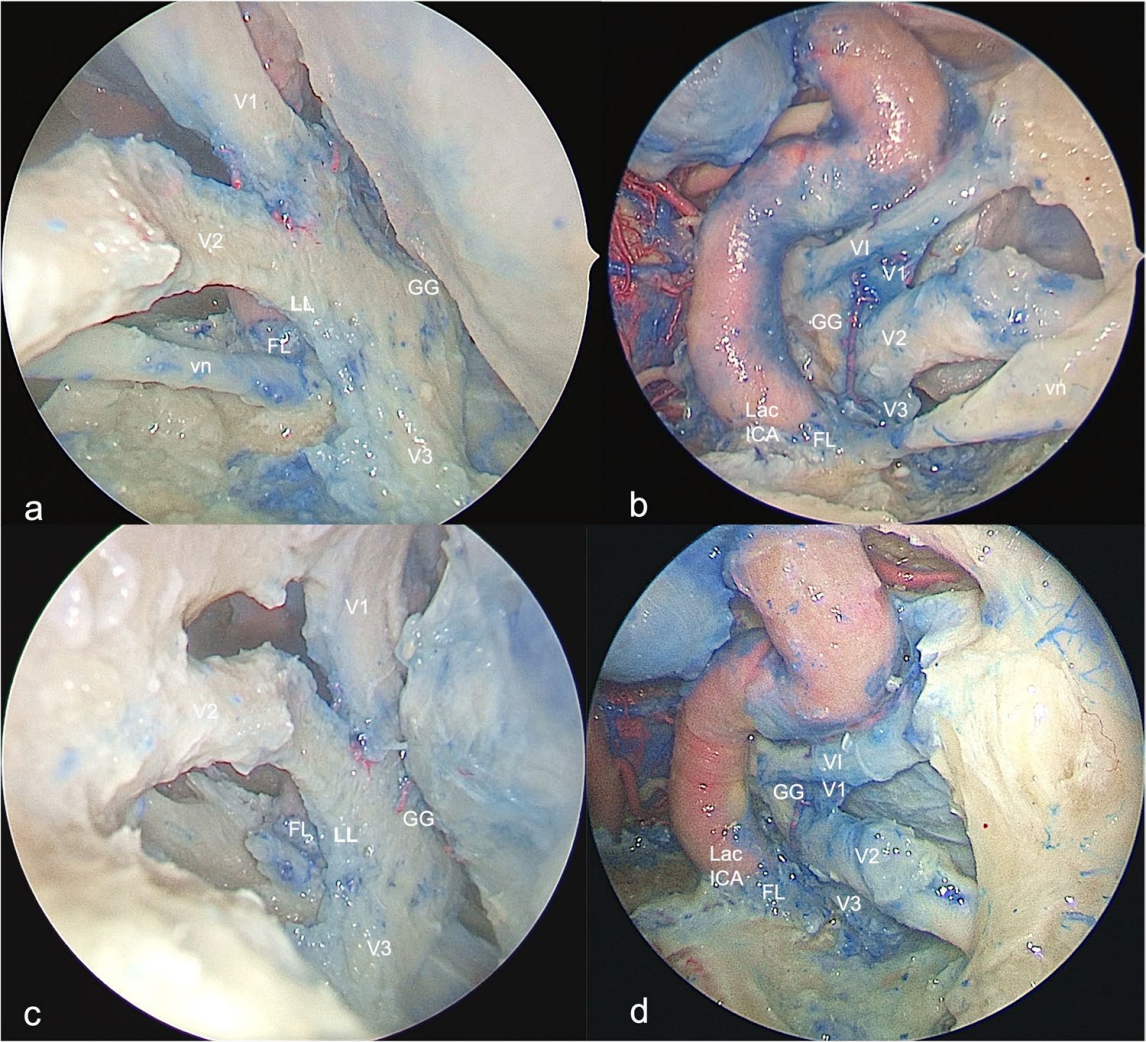
Exposure of the foramen lacerum region through endoscopic transorbital (**a**, **c**) and extended endonasal transclival (**b**, **d**) corridors before (**a**–**b**) and after (**c**–**d**) removal of the vidian nerve (left side). The lateral-to-medial trajectory provided by the transorbital approach through the anterolateral triangle represents a complementary surgical route to the medial-to-lateral trajectory provided by the endonasal corridor to the foramen lacerum region. (GG, gasserian ganglion; Lac ICA, lacerum internal carotid artery; CLIV, clivus; FL, foramen lacerum; vn, vidian nerve; LL, lateral loop; PLL, petro-lingual ligament)

**Fig. 6:**
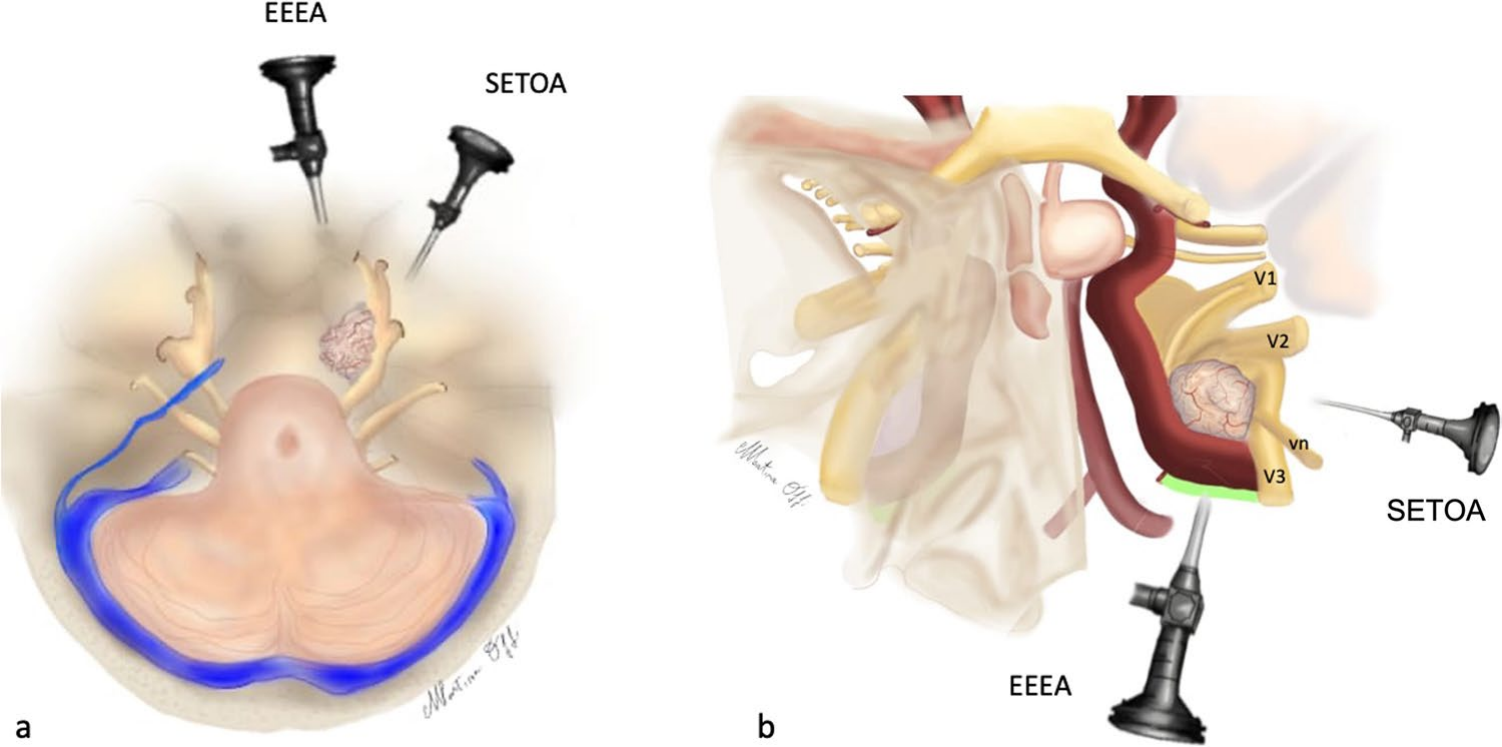
Artistic draws, axial (**a**) and coronal (**b**) views. Complementary extended endoscopic endonasal (EEEA) and superior eyelid endoscopic transorbital (SETOA) approaches to the lesion of lacerum foramen and medial aspect of Meckel’s cave

While SETOA to the middle cranial fossa can be considered a complementary route to the preauricular infratemporal and retrosigmoid approaches to access the lateral and posterior aspects of the MC[37], the new corridor explored via anterolateral triangle can be considered a complementary or alternative pathway — in selected patients according to the pathology and patients features — to the endoscopic endonasal transpterygoid approach to the anterior and medial aspects of this dural pocket, avoiding manipulation of the vidian nerve and with shorter working distance[1] (Figs. 5 and 6).

Table 2 summarizes the main pros and cons of the microsurgical transcranial anterolateral (fronto-temporo-orbito-zygomatic, FTOZ), extended endoscopic endonasal (EEEA), transpterygoid and endoscopic transorbital (SETOA) approaches in accessing the anteromedial aspect of the Meckel’s cave.

**Table 2:** Comparison between microsurgical fronto-temporo-orbito-zygomatic (FTOZ), extended endoscopic endonasal transpterygoid (EEEA) and endoscopic transorbital (SETOA) approaches to the antero-medial aspect of Meckel’s cave

Approach	Trajectory	Corridor	Key surgical landmarks to lacerum ICA	Advantages	Limitations
FTOZ	Anterolateral	- Anteromedial triangle of MCF	- Vidian nerve	––	- Full mobilization or transection of V3 and/or GG
		- Anterolateral triangle of MCF			- Retraction and manipulation of brain tissue, cranial nerves, and vessels
					- Risk of injury of lacerum ICA
					- Poor working angle
EEEA transpterygoid	Anteromedial	-Trans-maxillary Transpterygoid	- Vidian nerve—“Carotid sock”—Pterygoclival ligament	- Close view to the surgical field	- Degree of sphenoid sinus pneumatization
				- Avoiding crossing the plain of cranial nerves and vessels	- Transection of vidian nerve with related ophthalmic complications
				- More easy dissection between trigeminal nerve and the periosteal layer of dura mater	- Low risk of injury of lacerum ICA
				- Optimal angle of attack	- CSF leak risk
				- Abducens nerve under control	
SETOA	Anterolateral	- Anterolateral triangle of MCF	- Trigeminal lateral loop	- Close view to the surgical field	- Narrow corridor
			- Vidian nerve	- Absence of manipulation of brain tissue, cranial nerves and vessels	- Risk of injury of GG and trigeminal nerve branches which are superficial to the target area
				- Favorable working axis	- Risk of injury of lacerum ICA which is hidden by the lesion
				- Short working distance	
				- Abducens nerve under control	

*FTOZ*, fronto-temporo-orbito-zygomatic; *EEEA*, extended endoscopic endonasal approach; *SETOA*, superior eyelid trans-orbital approach; *MCF*, middle cranial fossa; *GG*, gasserian ganglion

### Key surgical landmarks

The knowledge of the exact localization of the ICA, both during the preoperative planning and intraoperatively, is crucial in the management of the skull base lesions close to the clivus and the adjacent areas, regardless of the type of surgical approach adopted.

Although more recently other surgical landmarks have been proposed to identify the lacerum segment of ICA via endoscopic endonasal route, such as the so-called carotid sock [28] and the pterygoclival ligament[44], because of its intracranial localization deep-seated in the ventral paramedian skull base beneath the floor of the sphenoid sinus and its relationship with several anatomical structures that represent the gateway to surgical approaches, i.e., the nasal cavity and maxillary sinus, or target areas, i.e., the cavernous sinus, the petrous apex, the Meckel’s cave, the foramen lacerum, and the petrous carotid — or simply encountered along the surgical corridor, i.e., the pterygopalatine fossa — the vidian nerve is considered the main surgical landmark in various operative procedures, both microsurgical and endoscopic, to the skull base[23, 33, 45].

The transorbital endoscopic approach through the drilling of the anterolateral triangle of the middle cranial fossa allowed us to expose the vidian nerve and follow its course until its disappearing behind V3 and after crossing the anterolateral surface of the lacerum segment of the ICA into the homonymous foramen; therefore, this nerve represents a key surgical landmark to the foramen lacerum and its content, even during transorbital endoscopic surgery.

Nevertheless, sometimes this nerve cannot be used as landmark because it is sacrificed during the operative procedure[1, 9, 22, 23, 51] or because its canal is involved by the lesion.

In this scenario, we identified the “lateral loop” of the trigeminal nerve[48] — the dural bridge between V2 and V3 — as valid alternative or additional constant and reliable landmark to the vidian nerve for identifying the lacerum ICA during SETOA, and we consider the maxillary branch a safe road map. As result, early identification of this landmark and its exposure after interperiosteal-dural dissection of the middle fossa allows the surgical exposure of the foramen lacerum, minimizing the risk of accidental injury to the lacerum ICA.

The foramina rotundum and ovale and the midsubtemporal ridge[48] represent key bony landmarks to identify the anterolateral triangle. Skeletonization of the vidian nerve starts by drilling just inferiorly to the foramen rotundum to identify the most anterior end of exposure of the homonymous nerve; then, it proceeds posteriorly by drilling the lower 180° of the vidian canal, as well-established technique to identify the lacerum segment of the ICA via EEEA[23], up to the foramen lacerum where the vidian nerve disappears behind V3. Lastly, the drilling along the inferior border of V2 up to the trigeminal lateral loop completes the exposure of the vidian nerve. At this point, the lacerum ICA is exposed between the vidian nerve inferiorly and the lateral loop of the trigeminal nerve superiorly.

The transorbital endoscopic route respects the principles of modern skull base minimally invasive techniques: flattening the skull base and using the extradural space to approach the target lesion while reducing brain retraction. Suero Molina et al. [43] in a recent literature review analyze the different surgical approaches to reach Meckel’s cave for tissue sampling of such indeterminate lesions. In this scenario, the endoscopic transorbital route may be considered a further option in the armamentarium of the neurosurgeons dealing with lesions involving not only the lateral but also the anteromedial aspect of Meckel’s cave.

Nevertheless, although SETOA provides several advantages, such as scar hidden within the eyelid crease, no temporalis muscle disruption, a rapid and small craniectomy, straight route to the MC with minimal brain retraction and without violating the cavernous sinus, sparing the vidian nerve avoiding the morbidities related to its injury, a mandatory consideration must be kept in mind when using SETOA: the width of the surgical corridor. This route, while providing wide visualization of the parasellar region, uses a narrow and single if compared to the binostril of the expanded endoscopic endonasal, surgical corridor which imposes limitations on surgical freedom and working angles.

### Limitations of this study

Pure anatomical studies have the common limitation related to the cadaveric specimens. The first limit is represented by the small number of specimens used. The properties of cadaveric tissues considerably differ from real anatomy: the consistency of the tissues, variability in size and pneumatization of the sphenoid sinus, the variability in size of the trigeminal ganglion, trajectory of the internal carotid artery and cranial nerves, and bone protuberances of the skull base should be considered. No quantitative data.

## Conclusion

The endoscopic transorbital approach via anterolateral triangle of the middle fossa can be considered a minimally invasive route complementary to the extended endoscopic endonasal to the anteromedial aspect of the Meckel’s cave and the foramen lacerum. The angle described by the origin of the maxillary and mandibular divisions of the trigeminal nerve from the gasserian ganglion, also known as trigeminal “lateral loop,” represents a valid surgical landmark to the lacerum segment of the ICA and the course of the maxillary division of the trigeminal nerve a safe road map to it. Nevertheless, as any new operative technique, a learning curve is required and feasibility in a clinical setting must be demonstrated.

## Data Availability

Data of the current original research are available from the corresponding author on reasonable request.

## Abbreviations

**SETOA**: Superior eyelid transorbital endoscopic approach
**EEEA**: Extended endoscopic endonasal approach
**ICA**: Internal carotid artery
**GG**: Gasserian ganglion
**MOB**: Meningo-orbital band
**MMA**: Middle meningeal artery
**CS**: Cavernous sinus
**FL**: Foramen lacerum
**PL**: Petrolingual ligament
**vn**: Vidian nerve
**pICA**: Petrous internal carotid artery
**FTOZ**: Fronto-temporo-orbito-zygomatic
**MC**: Meckel’s cave
